# Surgical treatment of a broken neuroplasty catheter in the epidural space: a case report

**DOI:** 10.1186/s13256-016-1064-7

**Published:** 2016-10-06

**Authors:** Tae Hyun Kim, Jun Jae Shin, Woo Yong Lee

**Affiliations:** 1 Department of Neurosurgery, Sanggye Paik Hospital, Inje University College of Medicine, Dongil-ro 1342, Nowon-gu, Seoul 01757 Korea; 2 Department of Anesthesiology, Sanggye Paik Hospital, Inje University College of Medicine, Dongil-ro 1342, Nowon-gu, Seoul 01757 Korea

**Keywords:** Lumbar spine, Lower back pain, Intervertebral disc herniation, Epidural catheter, Neuroplasty, Catheter breakage, Complications, Surgery

## Abstract

**Background:**

Percutaneous epidural neuroplasty with a Racz catheter is widely used to treat radicular pain caused by spinal stenosis or a herniated intervertebral disc. The breakage or shearing of an epidural catheter, particularly a percutaneous epidural neuroplasty catheter, is reported as a rare complication. There has been a controversy over whether surgical removal of a shorn epidural catheter is needed. Until now, only three cases related to sheared Racz neuroplasty catheters have been reported. We report a case of a neuroplasty catheter which completely broke when it was inserted into the epidural space, and compressed root symptoms were exacerbated by the broken catheter.

**Case presentation:**

A 68-year-old Asian man with leg pain and lower back pain caused by lumbar vertebral body 4 to lumbar vertebral body 5 intervertebral disc herniation and stenosis underwent percutaneous epidural neuroplasty. During the procedure, the epidural neuroplasty catheter was trapped in the left foraminal portion and broke. Our patient complained of left-side leg pain and numbness. Surgery performed to remove the broken catheter led to complete resolution of his leg pain and numbness.

**Conclusions:**

We report a rare case of catheter breakage occurring during epidural neuroplasty. We suggest surgical removal because the implanted catheter can aggravate a patient’s symptoms and lead to the development of neurologic deficits due to infection, fibrosis, or mechanical neural irritation.

## Background

Percutaneous epidural neuroplasty is increasingly performed in patients with back pain due to herniation of intervertebral discs (HIVD), spinal stenosis, or failed back surgery syndrome [1]. Complications of epidural neuroplasty include hematoma and infection in the epidural space, urinary and fecal dysfunction, sexual dysfunction, and paresthesia [2]. Other technical complications, including bending of the needle tip, penetration of the dura, nerve injury, subdural insertion, and catheter shearing, can also occur during the procedure [1, 3]. While epidural catheters can be routinely removed without any complications, in rare instances, neuroplasty catheter breakage can occur. The objective of this report was to present a rare case of a broken catheter which was treated surgically after percutaneous epidural neuroplasty and to review the unusual cases of epidural catheter breakage reported in the literature.

## Case presentation

**Fig. 1:**
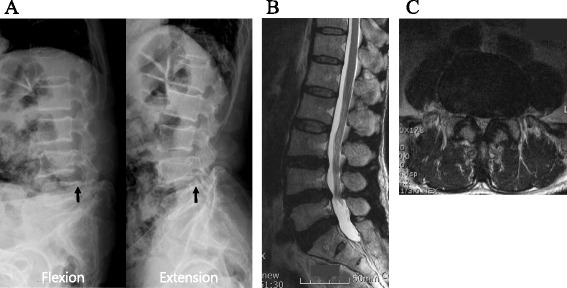
Preoperative radiographs and lumbar magnetic resonance imaging. **a** Dynamic plain radiographs showing degenerative spondylolisthesis of lumbar vertebral body 4 on lumbar vertebral body 5. The radiographic finding showed anterior slipping of lumbar vertebral body 4 on lumbar vertebral body 5 when the patient bent his back (*arrow*). An extension view shows the slight reduction of anterior slippage of lumbar vertebral body 4 on lumbar vertebral body 5 (*arrow*). **b** Sagittal view showing disc protrusion and stenosis of both foramen on lumbar vertebral body 4 and lumbar vertebral body 5. The dural sac was compressed ventrally and dorsally at lumbar vertebral body 4 and lumbar vertebral body 5 on a sagittal view of T2-weighted images. **c** Axial view showing disc protrusion, stenosis, and facet hypertrophy on lumbar vertebral body 4 and lumbar vertebral body 5. Narrowing of the spinal canal was found with both disc protrusion and ligamentum flavum hypertrophy

A 68-year-old Asian man presented to our out-patient department with lower back and bilateral leg pain for 1 year. The symptom was described as a “tingling sensation,” and the pain in both his thighs was aggravated by walking for more than 20 minutes. The diagnosis was neurogenic intermittent claudication, which was not relieved by analgesics, muscle relaxants, and other medications. Lumbar spine magnetic resonance imaging showed degenerative spondylolisthesis and severe degenerative central canal and bilateral foraminal stenosis (Fig. 1).

At first, we performed a transforaminal nerve root block, but his symptoms did not improve. We discussed his condition with a specialist from our anesthesiology department to formulate a plan for managing his symptoms. We decided to perform percutaneous epidural neuroplasty with a Racz catheter. If his symptoms and signs were not relieved with neuroplasty, a surgical procedure would be considered.

After obtaining our patient’s informed consent, we transferred him to our operation room, and he was placed in a prone position. Following sterile preparation and draping, we inserted a 16-gauge RX Coudé needle through his sacral hiatus. We performed epidurography with 10 ml of water-soluble contrast media. Before the epidural catheter was inserted, we checked its integrity by injecting 1 ml of sodium chloride to flush the catheter. The Racz neuroplasty catheter was inserted through the needle under continuous fluoroscopy. Undue resistance or abnormality did not occur during the insertion of the catheter and injection of materials. We injected 1500 units of hyaluronidase in 10 ml of normal saline. In addition, 9 ml of 0.125 % bupivacaine and 4 mg of dexamethasone were injected. After the injections, the anesthesiologist tried to pull out the catheter in the epidural space. During the procedure, our patient experienced severe burning pain on both buttock areas and suddenly moved his back and legs on the surgical table. We made him relax by injecting analgesics. Following this event, our patient complained of radiating pain on his left buttock. The anesthesiologist felt resistance while removing the catheter. Under fluoroscopy, the catheter could not be removed because it was trapped in the left foraminal portion of the vertebra. The distal tip of the sheared catheter was exposed and was palpable in the subcutaneous layer of the puncture site. As the anesthesiologist pulled the end portion of the catheter, the wire of the epidural catheter stretched out of shape and was not removed.

**Fig. 2:**
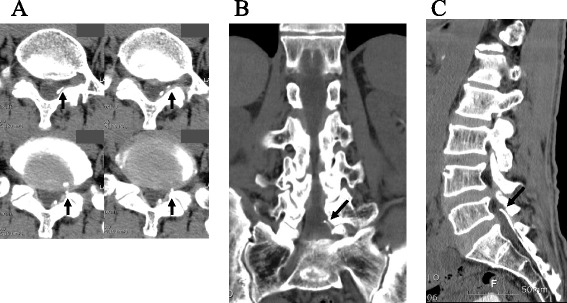
Lumbar spine three-dimensional computed tomography before catheter fragment removal. **a** Computed tomography axial view showing the compression of the left lumbar vertebral body 5 nerve root by a broken catheter tip (*arrow*). **b** Computed tomography coronal view showing that the catheter fragment tip is located at the left side of the lumbar vertebral body 5/sacral vertebral body 1 foraminal zone (*arrow*). **c** Computed tomography sagittal view showing the catheter fragment tip which spans from the sacral hiatus to the lumbar vertebral body 5/sacral vertebral body 1 level (*arrow*)

During the procedure, the epidural catheter was finally broken. The anesthesiologist continuously tried to remove the broken catheter in the coccyx but was unsuccessful. Subsequently, our patient experienced more severe radiating pain in his left leg. Lumbar spine three-dimensional computed tomography (CT) revealed that the epidural catheter tip was located at the left neural foraminal inlet at the lumbar vertebral body 5 (L5) to sacral vertebral body 1 (S1) level (Fig. 2). We explained the errant issue and its possible complications to our patient. We decided to perform surgical removal of the broken neuroplasty catheter with his consent. He was moved to our operating room and placed in a prone position under general anesthesia.

**Fig. 3:**
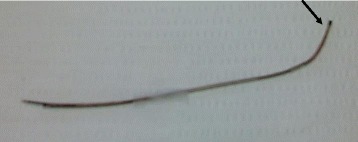
The epidural neuroplasty catheter. The broken epidural neuroplasty catheter was surgically removed. The proximal tip of the catheter was bent, but the tip sheath was not torn (*arrow*). The distal portion of the catheter was shorn, but there was no knotting or looping

A total punch laminectomy was performed, starting from the lower margin of the lamina of lumbar vertebral body 4 (L4). The catheter was confirmed to be present in the inlet area of the left L5 to S1 facet and was removed by retraction. The size of the retained catheter was 12 cm (Fig. 3). After identifying the nerve root of L4, we performed foraminotomy. The nerve root of L5 was trapped in the bony structure. The facet joints on L4 to L5 were totally removed. Subsequently, both L4 nerves were released. While the thecal sac and nerve root were retracted, discectomy was performed at the L4 to L5 level. We performed lumbar interbody fusion with cages and transpedicular screw fixations on L4 to L5 and then closed the surgical wound. Our patient’s symptoms subsided after the surgery. There was no evidence of infection at the operation site. During a 12-month postsurgical follow-up period, our patient experienced no symptoms and no neurological deficits.

## Discussion

In 1982, Racz *et al*. introduced percutaneous epidural neuroplasty, which relieves pain via local epidural adhesiolysis, neurolysis of vertebral nerve roots, and lavage of inflammatory mediators by injections of local anesthetics, corticosteroids, hyaluronidase, and hypertonic saline [1]. It is now considered a non-surgical treatment modality to relieve pain arising from spinal radiculopathy, back pain, and postsurgical pain syndrome [4]. Indications of epidural neuroplasty are lumbar disc herniation, lumbar stenosis, or radiculopathies with failed conservative management including physical therapy, chiropractic manipulation, exercises, drug therapy, and fluoroscopically directed caudal or transforaminal epidural injections [5–7]. Manchikanti *et al*. reported significant improvement in 82 % of patients with chronic low back and/or lower extremity pain after lumbar surgery with or without fusion [7]. Unsuccessful results or relative contraindications are shown in patients with pain originating in the facet joint or sacroiliac joint, heavy opioid use, uncontrolled psychiatric disorders, uncontrolled medical illness, or a history of or potential for adverse reactions to drugs such as lidocaine or betamethasone [7]. Although epidural neuroplasty is reported to be an effective method for treating low back pain, its potential complications include epidural bleeding, abscess, meningitis, paresthesia from nerve damage, dural tear, intravascular drug injection, hypotension, respiratory depression, and urinary and/or fecal incontinence [2]. Breakage or shearing of the catheter has been reported as a rare complication. In previous reports, the prevalence of epidural catheter breakage ranged from 0.002 to 0.04 % [8]. The necessity for surgical removal of a sheared epidural catheter has been controversial for many years. The current literature reports that sheared catheters are unlikely to cause neurologic deficits if they are sterile and inert [3].

In spite of these studies, several authors described the side effects of retained epidural catheters. Staats *et al*. reported the case of a retained epidural catheter which resulted in pain and weakness of the lower extremities 18 months after epidural anesthesia [9]. Blanchard *et al*. described a similar case of L3 radiculopathy 7 months after epidural anesthesia [10]. A recent report described a catheter fragment which was retained for 12 years and surgically removed after being incidentally identified [11]. In another case, migration of an epidural catheter was found 4 weeks after the occurrence of catheter breakage. The catheter was then surgically removed [12]. Three patients underwent surgical decompression under general anesthesia, resulting in complete relief of symptoms [10, 12, 13]; and in one case, the catheter was surgically retrieved under local anesthesia and fluoroscopic guidance because the patient was asymptomatic [11]. In all the cases described, the retained epidural catheters caused adhesion, granuloma formation, or fibrosis. In addition, there were several reports of immediate surgical removal of sheared epidural catheters. Lenox *et al*. reported the case of a 23-month-old child with a sheared catheter in the caudal epidural space [14]. The authors asserted that sequestrated catheters in young patients should be immediately removed surgically [11, 14].

In all the aforementioned cases, the breakage of catheters occurred during spinal anesthesia with an epidural catheter. Catheter shearing and its retention within the epidural space is one of the major complications which occur during neuroplasty procedures. The symptoms of compressed root may be exacerbated by a torn neuroplasty catheter. The Racz catheter is a wire-embedded catheter with a fine sheath around the wires. It has been specifically and strongly designed for neuroplasty. Nonetheless, there is a risk of shearing when the catheter is moved back and forth. If the length of the catheter is long, the epidural catheter may become kinked or bent. Similar to our case, the chance of a lodged catheter may increase with severe spinal degenerative changes, such as foraminal stenosis, spondylolisthesis, ligamentum flavum, or facet hypertrophy. In particular, if the patient’s position markedly changes during the procedure, the catheter may lodge on lumbar bony structures. To the best of our knowledge, three cases related to sheared Racz neuroplasty catheters have been reported [15–17]. Most reports were of asymptomatic cases of catheters which had been retained for a long time in the epidural space. Our case had a completely broken neuroplasty catheter while it was inserted into the epidural space, and the compressed root symptoms were exacerbated by the broken catheter.

The treatment strategy for retained epidural catheters has been controversial. It has been believed that it is safe to leave a fragment within the spinal canal if neurological symptoms or signs do not exist [3]. However, foreign bodies such as catheters retained within the spinal canal can potentially cause neurological deficits or clinical symptoms such as headache, inflammation, or migration. Recently, many authors have reported on the possibility of removing retained catheters. Manchikanti and Bakhit reported a catheter shear in which a torn piece was retained in the epidural space during percutaneous lysis of epidural adhesions [17]. The torn catheter could be removed with arthroscopy and forceps. We would like to consider the various methods to remove retained catheters on a case-by-case basis and to examine the necessity of local or general anesthesia.

In most cases, the broken or sheared catheters were retained in the epidural space. In an unusual case, a patient developed a severe headache when the broken catheter became trapped in the subarachnoid space. After a lumbar laminectomy and the removal of the trapped catheter from the subarachnoid space, the patient’s symptoms were relieved [18].

If the catheter breaks during its insertion or removal, we should inform the patient of the treatment options available and the complications which may arise. In particular, many authors recommend the removal of sheared catheters in children because of the possibility of neurological deficits [3, 13]. During the insertion of the catheter in the epidural space, special attention should be paid to prevent shearing or breaking of the catheter in patients with severe osteoarthritis, ossification of ligament flavum, or degenerative kyphotic deformity [18]. These degenerative structural changes may contribute to the development of problems similar to those seen in our case. Most cases of epidural catheter breakage have been associated with various factors, such as catheter kinking or knotting during its insertion or removal, partial shearing in supraspinous and interspinous ligaments, and faulty design of the catheter. Recommendations on preventing breakage of epidural catheters have been proposed, including meticulous physical examination, avoidance of faulty catheters, and avoidance of excessively long insertion lengths [18].

If it is difficult to remove the catheter, efforts to remove it should be discontinued after 15 to 30 minutes. It is important for the patient to be placed in the same position during the extraction of the catheter. Mitra and Fleischmann suggested various salvage techniques for removal of trapped epidural catheters, such as using slow continuous force and attempting removal after the injection of preservative-free normal saline through the catheter [3]. The use of a convex surgical frame and CT scan should be considered during removal to identify the etiology of entrapment.

In the present case, the patient experienced back pain, radiculopathy, and intermittent neurogenic claudication due to spinal stenosis and HIVD. We first considered the non-operative method of percutaneous neuroplasty to treat his clinical symptoms, but catheter breakage occurred during the procedure. Subsequently, we carefully discussed the errant event with him and decided to remove the sheared catheter via a surgical procedure under general anesthesia. In cases of difficulty in removing the catheter, we are careful not to cause stretching or breakage by applying excessive tension. It is also important to confirm the location of the catheter and evaluate the etiology using an imaging study such as CT. We do not recommend using a stainless steel hemostat or similar instruments for removing the catheter [19]. In our case, the anesthesiologist tried to remove the retained catheter by grasping it with a hemostat. However, this attempt caused the catheter to stretch and break. We recommend that if it is difficult to remove the catheter, gentle tension should be applied. Removal should be attempted several hours later, placing the patient in the lateral decubitus position and applying continuous tension on the catheter itself to remove it. In the case of difficult epidural catheter removal, the catheter should be removed without incident and with the tip intact after allowing the patient to relax for 3 hours [20]. The catheter could easily be removed without excess traction through back muscle relaxation [20]. The injection of normal saline and expansion of the catheter is one of many removal methods [21]. Another method is that a sterile Tuohy needle is passed over the epidural catheter and advanced to the epidural space, after which the Tuohy needle and lodged catheter are pulled out together [22]. However, this method could totally shear the catheter and compound the problem. For easy removal of the catheter, the patient is placed in the same position as that at the time of insertion to reduce the tensile strength. The lateral decubitus position is superior to the sitting position because gravity is removed [3]. The attachment of tape to the patient’s body with a pull of the catheter can easily remove the catheter by means of the patient’s flexion and extension movements [3].

We suggest surgery if breakage of the epidural catheter occurs. We found that there was a high possibility of neurological sequelae due to the compressed lesion aggravated by the retained catheter. Based on our case, breakage or shearing of the epidural catheter may possibly develop in patients with lumbar spondylolisthesis or foraminal lumbar stenosis combined with ligament and facet hypertrophy. If the patient’s position markedly changes, the catheter might become trapped or be difficult to remove, and the catheter might be lodged on the bony structure. In addition, if neurological deficits or clinical symptoms such as pain or numbness caused by a retained catheter occur, we suggest surgery to relieve clinical symptoms. This report will help clinicians consider the management options for retained broken catheters in patients with symptomatic herniated discs.

## Conclusion

The surgical removal of a sheared catheter after percutaneous epidural neuroplasty is necessary when the patient has symptoms of spondylopathy because the implanted catheter can aggravate the patient’s symptoms and lead to the development of neurologic deficits including infection, fibrosis, or mechanical neural irritation.

## Abbreviations

CT: Computed tomography
HIVD: Herniation of intervertebral discs
L4: Lumbar vertebral body 4
L5: Lumbar vertebral body 5
S1: Sacral vertebral body 1
